# Bubble‐Channeling Electrophoresis of Honeycomb‐Like Chitosan Composites

**DOI:** 10.1002/advs.202203948

**Published:** 2022-09-30

**Authors:** Bo‐Han Huang, Li‐Jie Chen, Yu‐Jie Chiou, Grace Whang, Yunkai Luo, Yichen Yan, Kung‐Hwa Wei, Ximin He, Bruce Dunn, Pu‐Wei Wu

**Affiliations:** ^1^ Department of Materials Science and Engineering National Yang Ming Chiao Tung University Hsinchu 300 Taiwan; ^2^ Department of Materials Science and Engineering UCLA Los Angeles CA 90095 USA

**Keywords:** chitosan, electrophoresis, polyethylene glycol, porous composites, silver nanoparticles, vertical channels

## Abstract

A chitosan composite with a vertical array of pore channels is fabricated via an electrophoretic deposition (EPD) technique. The composite consists of chitosan and polyethylene glycol, as well as nanoparticles of silver oxide and silver. The formation of hydrogen bubbles during EPD renders a localized increase of hydroxyl ions that engenders the precipitation of chitosan. In addition, chemical interactions among the constituents facilitate the establishment of vertical channels occupied by hydrogen bubbles that leads to the unique honeycomb‐like microstructure; a composite with a porosity of 84%, channel diameter of 488 µm, and channel length of 2 mm. The chitosan composite demonstrates an impressive water uptake of 2100% and a two‐stage slow release of silver. In mass transport analysis, both Disperse Red 13 and ZnO powders show a much enhanced transport rate over that of commercial gauze. Due to its excellent structural integrity and channel independence, the chitosan composite is evaluated in a passive suction mode for an adhesive force of 9.8 N (0.56 N cm^−2^). The chitosan composite is flexible and is able to maintain sufficient adhesive force toward objects with different surface curvatures.

## Graphical abstract

A chitosan composite with a vertical array of pore channels is fabricated via a bubble‐channeling electrophoretic deposition process. This unique honeycomb‐like microstructure demonstrates impressive mass transport behavior over that of commercial gauze. In addition, the chitosan composite shows an adhesive force of 9.8 N (0.56 N cm^−2^) in a passive suction mode and is flexible to accommodate different surface curvatures.

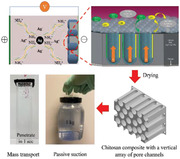

## 1 Introduction

Chitosan is a natural biopolymer known as a cationic polysaccharide and is recognized for its biocompatibility, antimicrobial ability, and biodegradability.^[^ ^1^ ^]^ In the literature, chitosan and its composites have been studied in food packaging, drug release, chemical absorption, and wound dressing.^[^ ^2^ ^]^ In addition, chitosan has attracted considerable attention as a scaffold because its biodegradability, pH sensitivity, and mechanical strength could be readily tailored to address specific applications.^[^ ^3^ ^]^ Previously, the formation of chitosan thin film via electrophoretic deposition (EPD) has been employed for coating purposes.^[^ ^4^ ^]^ The EPD is a process in which charged colloids are driven toward the electrode with opposite polarity for film deposition. In an acidic solution, the chitosan is protonated to carry positive surface charges and thus migrates to the cathode under an electric field. Simultaneously, water electrolysis is occurring on the cathode that produces H_2_ bubbles with the release of OH^−^. As a result, the localized pH value near the cathode is increased which deprotonates the chitosan for film formation. It is noted that a typical EPD‐derived chitosan film reveals poor uniformity and many surface cavities left by the entrapped H_2_ bubbles. Consequently, the resulting chitosan film often lacks sufficient mechanical strength to be free‐standing.

To improve the mechanical strength for a robust “free‐standing film” chitosan via EPD, a variety of chitosan composites have been explored. For example, Wang et al. used H_2_O_2_ and 1‐ethyl‐3(3‐dimethylaminopropyl)carbodiimide during EPD to produce a chitosan/gelatin/Ag nanoparticles (NPs) composite with sufficient surface uniformity for biomedical purposes.^[^ ^5^ ^]^ Pan et al. fabricated a carboxylated chitosan/Ag NPs composite by in situ Ag oxidation and reduction during EPD.^[^ ^6^ ^]^ However, those samples are dense and solid with negligible pores or cavities. In addition, there are efforts to fabricate chitosan composites with porous structures. For example, Bonetti et al. prepared mixtures of chitosan and Nb‐doped bioactive glass via EPD for bone regeneration.^[^ ^7^ ^]^ Ghalayani Esfahani et al. synthesized chitosan/bioactive glass scaffolds with a hierarchical microchannel architecture for bone regeneration.^[^ ^8^ ^]^ However, their morphologies are relatively disordered with many native defects. Therefore, it is critical to develop an effective route for the fabrication of free‐standing chitosan composite with well‐arranged pore channels and specific pore sizes.

To date, a variety of synthetic routes including chemical reduction, electrochemical reduction, and photon irradiation have been adopted to synthesize Ag NPs for biomedical, electrical, and optical applications.^[^ ^9^ ^]^ In particular, for biomedical use, the “green” synthesis of Ag NPs becomes increasingly important. Previously, Ahmad et al. combined chitosan, polyethylene glycol (PEG), and Ag^+^ to form a metallopolymer, and both the chitosan and PEG served as the stabilizer.^[^ ^10^ ^]^ It is noted that the chitosan and PEG are ideal candidates for “green process” because the chitosan is able to chelate the Ag^+^ ions with its amine and hydroxyl groups, and both chitosan and PEG are capping agents for Ag NPs due to the electrostatic interaction between the positively‐charged Ag NPs and the hydroxyl groups in chitosan and PEG.^[^ ^10^, ^11^ ^]^ Thus, we recognize the unique opportunity in combining chitosan, PEG, and Ag^+^ ions for EPD because both the chitosan and Ag NPs could be codeposited on the cathode while the PEG could serve as a stabilizer to inhibit parasitic H_2_ bubble formation.

In this work, we adopted the EPD to fabricate a chitosan composite (chitosan/PEG/Ag_2_O‐Ag NPs) with a unique honeycomb‐like microstructure in which independent pore channels with diameter of 488 µm were aligned vertically. The PEG functioned as a reducing agent and a stabilizer during the EPD, and helped to improve the hydrophilicity of chitosan composite while the Ag_2_O and Ag NPs were incorporated to provide antibacterial activity. To evaluate its potential application in wound dressing, experiments including water uptake, Ag releasing profile, and mass transport were conducted. In addition, the chitosan composite was also explored for adhesive applications such as object manipulation and negative pressure wound therapy due to its unique structural integrity and excellent pore independency.

## 2 Results and Discussion

### 2.1 Fabrication of Chitosan Composite

**Figure 1:**
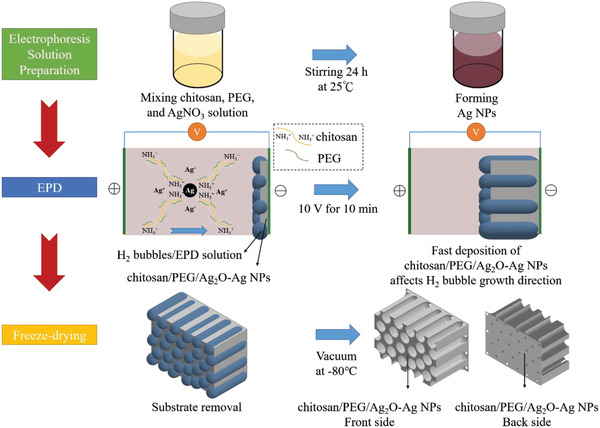
The schematic of processing steps involved for the fabrication of chitosan composite.

The chitosan composite was fabricated by first mixing chitosan, PEG, and silver nitrates to form an aqueous electrophoresis solution, followed by an EPD process in which the water electrolysis at the cathode induced the precipitation of chitosan and PEG, in conjunction with the formation of Ag_2_O and Ag NPs. With a proper combination of chemical composition and EPD parameters, a honeycomb‐like microstructure with independent vertical pore channels was formed in the resulting chitosan composite. **Figure** **1** displays the schematic for the processing steps involved. As shown, in an acidic solution, the chitosan is protonated to carry positive surface charges and thus migrates to the cathode under an externally imposed electric field. Simultaneously, water electrolysis is occurring at the cathode producing H_2_ bubbles with the release of OH^−^. As a result, the localized pH value near the cathode is increased which deprotonates the chitosan for film formation.

We also prepared control samples to distinguish the effect of individual constituents on the EPD process. The composition for electrophoresis solutions and their respective denotation for the resulting composite are listed in **Table** **1**.

**Table 1:** The composition of electrophoresis solution under study and their respective denotation for the resulting composite scaffolds. The chitosan is also denoted as the “CHI”

ID	Chitosan [%] [w v^−1^]	PEG [%] [w v^−1^]	AgNO_3_ [× 10^−3^ m]	H_2_O_2_[%] [w v^−1^]
**pristine chitosan**	1.43	0	0	0
**CHI/H_2_O_2_**	1.43	0	0	2.2
**CHI/PEG/H_2_O_2_**	1.43	10	0	2.2
**1Ag**	1.43	10	4.3	2.2
**3Ag**	1.43	10	12.9	2.2
**5Ag**	1.43	10	21.5	2.2

**Figure 2:**
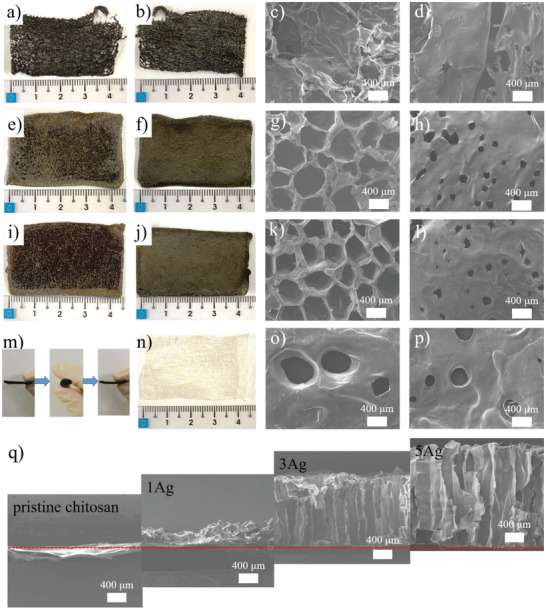
The photographs of 1Ag in a) front side and b) back side, as well as top‐view SEM images in c) front side and d) back side. The photographs of 3Ag in e) front side and f) back side, as well as top‐view SEM images in g) front side and h) back side. The photographs of 5Ag in i) front side and j) back side, as well as top‐view SEM images in k) front side and l) back side. The photographs of m) 5Ag undergoing a bending action and n) pristine chitosan. The top‐view SEM images of pristine chitosan in o) front side and p) back side. q) The cross‐sectional SEM images for pristine chitosan, as well as 1Ag, 3Ag, and 5Ag.

**Figure** **2** displays the photographs and top‐view scanning electron microscope (SEM) images of 1Ag, 3Ag, and 5Ag. The photographs for both front side (facing the electrophoresis solution) and back side (facing the working electrode) of 1Ag are exhibited in Figure 2a,b. Apparently, the 1Ag lacked structural integrity after detachment from the substrate, and many large through‐holes were observed. Figure 2c displays the SEM image of 1Ag on the front side. As shown, the surface morphology appeared rough and tattered. The back side of 1Ag, shown in Figure 2d, revealed a filmy and broken surface. In contrast, for the photographs of 3Ag shown in Figure 2e,f, the sample demonstrated a distinct morphology in which the front side contained many pores but the back side appeared smooth. The corresponding SEM image on the front side, shown in Figure 2g, revealed a distinct porous microstructure with a wide pore size distribution of 130–660 µm. Interestingly, its back side SEM image, shown in Figure 2h, maintained a surface with smaller pores whose size distribution was 60–230 µm. A similar morphology was observed for 5Ag shown in Figure 2i,j, in which the front side exhibited a uniform and porous microstructure whereas the back side appeared flat and smooth. The corresponding front side SEM image is displayed in Figure 2k. As shown, the pore size was uniform with a narrower distribution in 330–500 µm. The back side SEM image, shown in Figure 2l, displays a similar morphology to that of Figure 2h, and its pore size was in the range of 60–240 µm. Furthermore, the 5Ag was structurally robust as its increased thickness and homogeneous nature rendered it bendable in a reversible manner, as shown in Figure 2m. In contrast, the pristine chitosan, shown in Figure 2n, exhibited a porous and rumpled appearance with identical morphology for both front side and back side. The SEM images for pristine chitosan are displayed in Figure 2o,p for front side and back side, respectively. Apparently, their morphologies were similar in that solid chitosan skeletons were observed with scattered holes left by the entrapped H_2_ bubbles prohibiting the deposition of chitosan. This morphology was often observed in chitosan derived from EPD.^[^ ^12^ ^]^

The cross‐sectional SEM images for pristine chitosan, as well as 1Ag, 3Ag, and 5Ag are displayed in Figure 2q. These images were aligned with their respective working electrode so their deposit thickness and morphology were compared directly. Apparently, the pristine chitosan demonstrated a rough microstructure with a thickness of 90 µm. As stated earlier, its microstructure was affected by the interference of H_2_ bubbles that were moving, merging, and detaching from the deposit. For 1Ag, it exhibited a similar morphology to that of pristine chitosan but its thickness was increased considerably to 450 µm. For 3Ag and 5Ag, the morphologies demonstrated a notable transformation from the random distribution of pores to pore channels that were vertically aligned. In addition, these pore channels extended across the entire composite. It is noted that there appeared a thin film with a thickness of 5–10 µm near the back side, which was formed by the instant chitosan deposition at the very beginning of EPD. As a result, there appeared two distinct morphologies on the front side and back side. The thickness for 5Ag was around 2000 µm, a value that was greater than that of 3Ag whose thickness was around 1700 µm. This confirmed again that the 5Ag revealed the highest deposition rate among the samples under study. It is noted that for Ag ion concentration above 5Ag, the interactions between Ag^+^, Ag NPs, chitosan, and PEG became so intense that the mixture transformed to a semigel‐like state in 10 min. Consequently, the EPD became rather unstable, resulting in a poorly‐structured composite with many defects.

It is noted that the effect of ice formation during freeze‐drying was studied and determined to be negligible in affecting the desirable porous structure in chitosan composites. The detailed results and discussion are provided in Figure S1 in the Supporting Information. We also carried out additional rheology analysis to measure the viscosity of the electrophoresis solution. We found out that the viscosity for 5Ag was relatively stable so its EPD was able to proceed at a constant rate which facilitated the formation of vertically‐aligned pores. The detailed rheological studies are provided in the Supporting Information (Figure S2, Supporting Information). In our electrophoresis solution, each constituent plays its unique role during the EPD process. For example, the concentration of Ag^+^ and chitosan is critical in the resulting deposition rate and microstructure. The role of H_2_O_2_ is to remove excess H_2_ bubbles so the bubble‐channeling effect could be maintained. The PEG acts as a stabilizer to slow down the water electrolysis reaction. In our electrophoresis solution, we have optimized individual constituents and their results are displayed in Figure 2. Additional discussion on the chemical constituents and their interactions are provided in Figure S3 and Table S1 in the Supporting Information.

**Figure 3:**
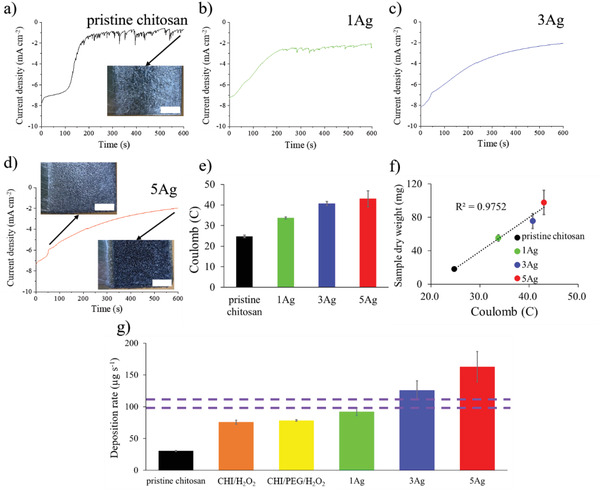
The current profiles during EPD for a) pristine chitosan, b) 1Ag, c) 3Ag, and d) 5Ag. The inset is their photographs. e) The corresponding coulomb charge. f) The sample dry weight versus coulomb charge. g) The deposition rate for different samples.

**Figure** **3** displays the current profiles during the EPD of pristine chitosan, as well as 1Ag, 3Ag, and 5Ag. As shown in Figure 3a, the pristine chitosan revealed a pattern in which the electrophoresis current began at −7 mA cm^−2^, and was quickly reduced to −1.5 mA cm^−2^ after 100 s. This reduction in current with increasing electrophoresis time was expected because, with simultaneous deposition of chitosan and formation of H_2_ bubbles from water electrolysis, the working electrode became more resistive. As a result, the EPD of chitosan became more difficult, leading to a decreasing current. In addition, there appeared considerable current fluctuations caused by the sudden release of adsorbed H_2_ bubbles after 200 s. From this current profile, it could be inferred that the microstructure of EPD‐derived chitosan would not be homogeneous due to frequent current fluctuation.^[^ ^13^ ^]^ The inset in Figure 3a displays the photograph of chitosan film on the working electrode. As expected, the surface appeared to be rather rough with the presence of many through‐holes and cavities.

Figure 3b displays the current profile for 1Ag. Interestingly, as compared to that of pristine chitosan, the current profile became relatively smoother, suggesting undesirable H_2_ bubble outburst was moderately subdued. In addition, the magnitude of current was consistently larger than that of pristine chitosan. We rationalized that the increased deposition rate for 1Ag hindered the movement and merge of H_2_ bubbles because those freshly‐produced H_2_ bubbles were likely to be entrapped separately, resulting in a smaller current fluctuation. In contrast, for both 3Ag and 5Ag, their current profiles, shown in Figure 3c,d, became much smoother with a negligible current fluctuation. At the same time, we recorded a much faster deposition rate as the resulting composites were significantly thicker than those of pristine chitosan and 1Ag. Moreover, the current profile clearly suggested a two‐stage electrophoresis process whose representative photographs (from 5Ag) are displayed in the inset of Figure 3d. In the first stage ranging from 0 to 50 s, the sample surface exhibited a homogeneous morphology with uniform bubbles. In the second stage ranging from 50 to 600 s, the sample revealed the presence of uniform pores. The overall thickness for 5Ag was 2 mm, and the EPD process was videotaped with the bubble entrapping phenomenon ((Video S1, Supporting Information).

To elucidate the cause responsible for structural transformation from the pristine chitosan (random) to 5Ag (vertical channel), the total coulomb charge during EPD for our samples was obtained. As shown in Figure 3e, the coulomb charge was increased with increasing Ag^+^ concentration. The comparison between sample dry weight and coulomb charge is displayed in Figure 3f. Apparently, there appeared a positive correlation, indicating the increase of Ag^+^ concentration led to a larger coulomb charge and a greater sample weight. To further clarify the effect of deposition rate on the microstructure of chitosan composite, we conducted the EPD with different combinations of constituents. Their resulting deposition rates are displayed in Figure 3g. The deposition rate is defined by the following formula

(1) $$\begin{array}{ccc} \mathrm{deposition}\;\mathrm{rate}\;(\mu g\;s^{-1}) & = & \mathrm{sample}\;\mathrm{dry}\;\mathrm{weight}\;\left(\mu g\right)/\\ & & \mathrm{deposition}\;\mathrm{time}\;\left(s\right) \end{array}$$

It is noted that both 3Ag and 5Ag demonstrated a desirable microstructure, and their deposition rates were also the highest among all electrophoresis solutions under study. Apparently, the formation of a thick composite required a high deposition rate. The critical deposition rate was suggested to be above 100 µg s^−1^ (purple dash line). In short, the sample with more Ag^+^ concentration engendered a faster deposition rate, and thus the H_2_ bubbles were less likely to form a barrier layer.

**Figure 4:**
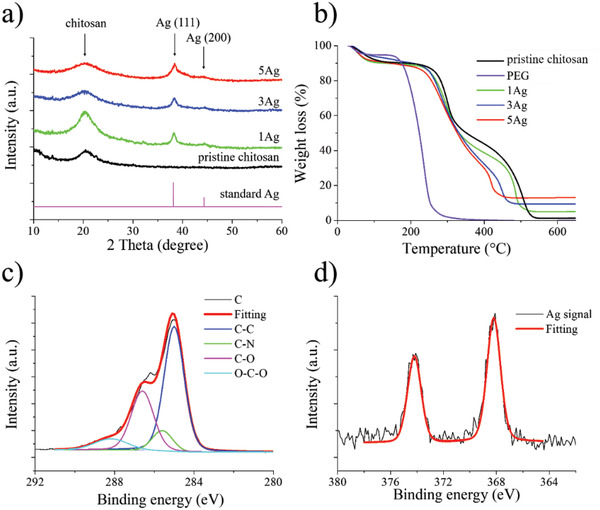
a) The XRD diffraction patterns for pristine chitosan, 1Ag, 3Ag, and 5Ag, as well as standard fcc Ag. b) The TGA profiles for pristine chitosan, 1Ag, 3Ag, and 5Ag. The 5Ag XPS profiles of c) C(1S) and its fitting curve and d) Ag(3d) and its fitting curve.

**Figure** **4**a displays the XRD patterns for pristine chitosan, 1Ag, 3Ag, and 5Ag, as well as the standard Ag (JCPDS: 04–0784) for comparison purpose. As shown, the pristine chitosan demonstrated a characteristic diffraction signal at 20.2°, which was caused by the crystalline chitosan *α* phase.^[^ ^14^ ^]^ For 1Ag, 3Ag, and 5Ag, there appeared additional diffraction signals at 38.1° and 44.3°, and they were attributed to the (111) and (200) planes of fcc Ag. Its grain size, estimated from the (111) plane using the Scherrer equation, was 8.9, 7.1, and 4.1 nm for 1Ag, 3Ag, and 5Ag, respectively. These values were consistent with what we observed in the TEM images shown in Figure S4 in the Supporting Information. It is noted that the formation of Ag NPs occurred during the preparation of electrophoresis solution.

Figure 4b displays the TGA profiles for pristine chitosan and PEG, as well as 1Ag, 3Ag, and 5Ag. Apparently, the pristine chitosan revealed a pattern in which a dehydration step occurred under 200 °C for a weight loss of 10%, followed by the initiation of pyrolysis for temperatures between 250 and 350 °C. At temperatures above 400 °C, the decomposition of chitosan was complete with a negligible residual weight. For PEG, the TGA profile demonstrated a dehydration step below 200 °C, followed by a rapid decomposition between 200 and 250 °C. For 1Ag, 3Ag, and 5Ag, their TGA profiles exhibited similar patterns to that of pristine chitosan but were slightly compromised in thermal stability. This was attributed to the incorporation of PEG that decomposed earlier than chitosan. From TGA profiles, the residual Ag amount for 1Ag, 3Ag, and 5Ag was 5%, 9.4%, and 12.9%, respectively.

The chemical constituents in chitosan composite were validated by FT‐IR. Detailed analysis is provided in Figure S5 in the Supporting Information. The chemical nature of C and Ag in 5Ag was investigated by X‐ray Photoelectron Spectroscopy (XPS), and the resulting XPS profiles are displayed in Figure 4c,d. Figure 4c displays the C(1s) profile and its fitting curves. It is noted that the signal at 284.8 eV was associated with the C–C and C–H bonds in chitosan and PEG,^[^ ^15^ ^]^ and it was the predominant peak in chitosan. In addition, the signal at 285.6 eV was due to the C–N bond from the glucosamine group of chitosan.^[^ ^15^ ^]^ For the signal at 286.7 eV, it was caused by the C–O and C–OH bonds in chitosan and PEG, and it was also the predominant signal in PEG.^[^ ^15^ ^]^ For the signal at 288.2 eV, it was due to the O–C–O bond from chitosan.^[^ ^15^ ^]^ Figure 4d displays the Ag(3d) profile and its fitting curves. The signals at 368.2 and 374.2 eV represented the Ag(3d_5/2_) and Ag(3d_3/2_), respectively. The XPS profile indicated the presence of metallic Ag instead of Ag^+^. It is noted that the analysis was conducted for the sample at the back side.

**Figure 5:**
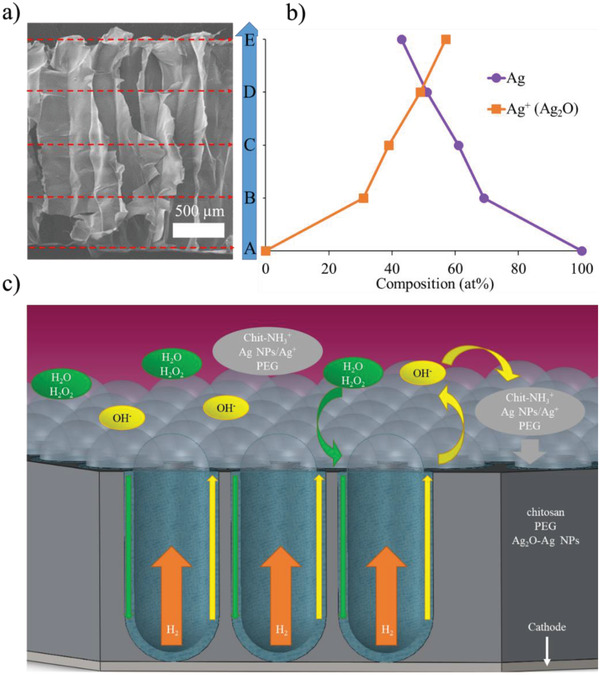
a) The cross‐sectional SEM image of 5Ag and the positons for XPS analysis. b) The corresponding XPS Ag(3d) analysis of Ag and Ag_2_O. c) A schematic of chemical reactions and mass transports occurring during EPD.

To understand the formation mechanism for the chitosan composite, the cross‐sectional XPS analysis of 5Ag was conducted, and the resulting Ag(3d) profile is displayed in **Figure** **5**a. The XPS signals were collected from the positions starting at 0 mm (A) to 2 mm (E) with 0.5 mm distance apart. This represented the composite formation process from the very beginning to the end. Position A represented the back side of the composite whereas position E represented the front side of the composite. The XPS fitting result of Ag(3d) is displayed in Figure 5b. As shown, only metallic Ag was observed in position A. However, with increasing height, the signal of Ag^+^ (from Ag_2_O) became stronger, with a larger Ag_2_O/Ag ratio (detailed XPS fitting curves are displayed in Figure S6 in a Supporting Information. It is noted that our electrophoresis solution contained both Ag^+^ and Ag NPs. As a result of strong interactions such as the chelating effect and electrostatic attraction between the Ag^+^/Ag NPs and the amine groups or hydroxyl groups of chitosan and PEG,^[^ ^11^, ^14^, ^16^ ^]^ once the concentration of Ag^+^ was sufficient (3Ag and 5Ag), the molecular chains of chitosan were strongly intertwined and became an extensive polymer network.^[^ ^17^ ^]^ In addition, the Ag^+^, Ag NPs, and protonated chitosan carried positive charges, which engendered a cohesive movement toward the electrode with a negative polarity during EPD. At the beginning of EPD process, there was a parasitic water electrolysis reaction occurring on the cathode surface that produced H_2_ bubbles, in conjunction with the release of OH^−^ and the increase of localized pH, as well as the removal of H_2_ bubbles by H_2_O_2_ and the reduction of Ag^+^ at the cathode.^[^ ^18^ ^]^ In addition, the OH^−^ was able to render the deposition of chitosan and the formation of Ag_2_O which led to a fast deposition rate. The detailed reactions steps are provided below

(2) $$\mathrm{Water}\;\mathrm{electrolysis}\;\mathrm{on}\;\mathrm{cathode}:2H_{2}O+2e^{-}\to H_{2}+2{\mathrm{OH}}^{-}$$

(3) $$\mathrm{Removal}\;\mathrm{of}\;\mathrm{hydrogen}\;\mathrm{bubbles}:H_{2}O_{2}+H_{2}\to 2H_{2}O$$

(4) $$\mathrm{Silver}\;\mathrm{reduction}:{\mathrm{Ag}}^{+}+e^{-}\to \mathrm{Ag}$$

(5) $$\begin{array}{ccc} & & \mathrm{Chitosan}\;\mathrm{deposition}:\;\mathrm{Chitosan}-{\mathrm{NH}}_{3}^{+}+{\mathrm{OH}}^{-}\\ & & \;\to \mathrm{Chitosan}-{\mathrm{NH}}_{2}+H_{2}O \end{array}$$

(6) $$\begin{array}{ccc} & & \mathrm{Formation}\;\mathrm{of}\;\mathrm{silver}\;\mathrm{oxide}:2{\mathrm{AgNO}}_{3}+2{\mathrm{OH}}^{-}\\ & & \;\to {\mathrm{Ag}}_{2}O+H_{2}O+2{\mathrm{NO}}_{3}^{-} \end{array}$$

We rationalized that as the chitosan composite was growing at a fast pace, the growth direction of H_2_ bubbles was limited to be vertical as any side movement of H_2_ bubbles was unlikely, as depicted in Figure 5c. In addition, the OH^−^ group produced from the water electrolysis diffused toward the electrophoresis solution along the side walls of H_2_ bubbles, resulting in the preferential deposition of chitosan, Ag_2_O, and chelated Ag NPs. Simultaneously, both water and H_2_O_2_ diffused to the electrode along the same H_2_‐bubble side walls to participate in the water electrolysis and the partial reduction of H_2_ bubbles. Therefore, for 3Ag and 5Ag, the deposition rate of chitosan/PEG/Ag_2_O‐Ag NPs and the growth rate of H_2_ bubbles were nicely balanced, and thus a uniform growth toward the electrophoresis solution was realized, resulting in independent pore channels that were properly aligned. It is noted that upon the completion of EPD, the chitosan composite demonstrated impressive continuity and robustness so it was able to be detached from the substrate in a wet state for subsequent freeze‐drying step. The detailed formation process (5Ag) was recorded as shown in Video S2 in the Supporting Information. Further experiments were carried out to validate the formation mechanism in which the pore channel diameter was inversely proportional to the deposition rate, and the results are shown in Figures S7–S10 in the Supporting Information.

### 2.2 Experiments for Water Intake and Mass Transport

**Figure 6:**
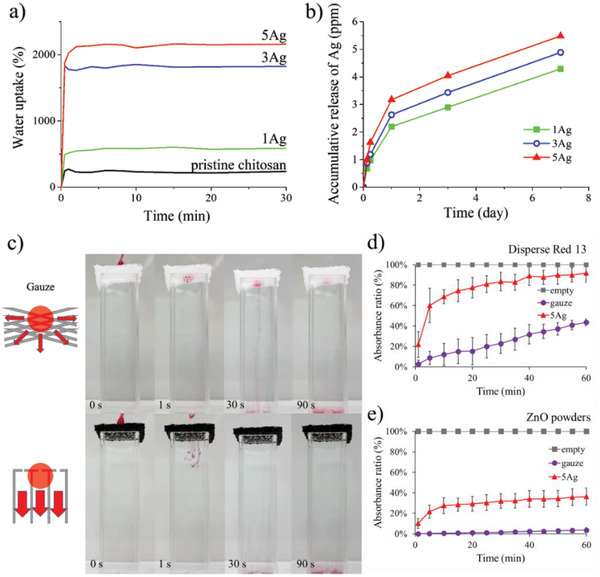
a) The water‐intake profiles for pristine chitosan, 1Ag, 3Ag, and 5Ag. b) The Ag release profiles for 1Ag, 3Ag, and 5Ag. c) The photograph of a droplet of red ink across gauze and 5Ag, respectively. The UV–vis absorbance ratio of d) Disperse Red 13 and e) ZnO powders across gauze and 5Ag, as well as the control sample (empty), respectively.

To explore potential applications in tissue engineering and wound dressing, additional experiments were carried out. **Figure** **6**a displays the amount of water uptake as a function of time for pristine chitosan, as well as 1Ag, 3Ag, and 5Ag. As shown, the pristine chitosan demonstrated a water uptake of 250% in 30 s. This moderate amount was due to its lack of excess pores so the available space for water retention was relatively limited. For 1Ag, the amount of water uptake was increased to 550% in 30 s. It is because the 1Ag exhibited a 3D porous microstructure allowing more spaces for water retention (from the SEM images in Figure 2). For 3Ag and 5Ag, since they exhibited microstructures with vertically aligned pore channels, the resulting water uptake was increased significantly to 1800% and 2100%, respectively. It is because their improved thickness and porosity was able to provide a large capacity to retain water. It is noted that our water uptake is significantly larger than those of chitosan and its composites reported in earlier literature. For example, Karimi et al. synthesized a porous composite of chitosan and alginate with a water uptake of 667%.^[^ ^19^ ^]^ In addition, Asadpour et al. prepared a composite of chitosan and gelatin with a water intake of merely 84–90%.^[^ ^20^ ^]^

For practical applications, the release of Ag is an issue to be concerned with. Figure 6b displays the Ag release profile for our samples in PBS. As shown, these samples revealed a similar pattern in which a rapid Ag release was recorded during the first 24 h, followed by a slower release. In addition, the severity of Ag release was increased with increasing Ag loading in the chitosan composite, a pattern that was reasonably expected. According to earlier literature, any material with a released Ag concentration of 0.2 ppm and above was able to demonstrate the desirable antibacterial effect.^[^ ^21^ ^]^ From these profiles, our chitosan composite demonstrated both instant Ag release against bacteria growth and gradual release for long‐term wound protection.

To validate its unique advantage for facile mass transport (for poential use in feeding drugs from outside of the wound dressing), we conducted a simple demonstration in which a typical gauze (about 2000 µm in thickness) and a 5Ag (2000 µm in thickness) were positioned atop a water‐containing cuvette in a wet state and a 10 µL droplet of red ink was dropped and diffused across the gauge and 5Ag, respectively. The red ink represented the drug so its movement was visible. Figure 6c displays the photographs of samples at different times. The microstructure of gauze was consisted of intertwining gauze wires so its pores were randomly distributed. As a result, the ink diffused across the gauge in a 3D random walk and it took 90 s to complete. In contrast, for 5Ag it took less than 30 s for the ink to diffuse through. This was due to the vertically aligned channels that facilitated the mass transport in a straight line. The complete video for this experiment is provided in Video S3 in the Supporting Information.

We also attempt to quantify the mass transport via UV–vis analysis on chemicals such as Disperse Red 13 and ZnO powders. It is noted that the Disperse Red 13 is a common dye used in optical‐related applications because it could be readily detected from UV–vis.^[^ ^22^ ^]^ For Disperse Red 13 shown in Figure 6d, the 5Ag exhibited a decent mass transport behavior as compared to that of gauze. The ZnO powders are often prepared in a suspension to provide Zn in wounds for healing purpose.^[^ ^23^ ^]^ As shown in Figure 6e, the 5Ag revealed an impressive mass transport behavior whereas the gauze behaved otherwise due to its tortuous transportation pathways. In short, our chitosan composite demonstrated a perfect balance in microstructure that led to the desirable combination of wound dressing and drug delivery from outside.

### 2.3 Experiments for Passive Suction

**Figure 7:**
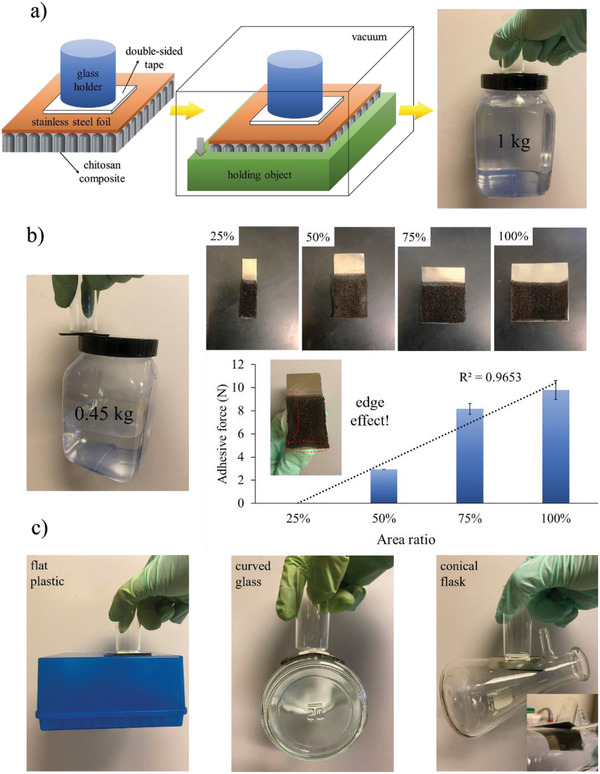
a) The experimental setup for adhesive test and the photograph of 1 kg loading for 5Ag. b) The photograph of adhesive force of 0.45 kg loading for 5Ag with a partial contact and the size dependence of adhesive force. c) The demonstration of adhesive force for 5Ag on different surfaces.

To further explore the unique structural characteristic of chitosan composite, we evaluated our sample in a passive suction mode as shown in **Figure** **7**. A passive suction cup requires a desirable structural integrity for intimate physical contact so negative pressure is maintained for loading bearing of the attachment. It is noted that the chitosan composite (5Ag) contained individual pore channels that acted like independent suction cups. Figure 7a displays the experimental setup for the adhesive test. The 5Ag was prepared on a stainless steel foil in a wet state (5 × 3.5 × 0.2 cm^3^), and the stainless steel foil was fastened by a double‐sided tape and a handler. Afterward, this sample was subjected to −80 kPa for 5 min for subsequent adhesive test. The photograph in Figure 7a also shows the adhesive performance for 1 kg loading. In Figure 7b, we validated that the pore channels in 5Ag were independent so the sample still showed impressive adhesive ability despite it was in partial physical contact with the attachment. To quantify the relationship between the contact area and the maximum adhesive force, we fabricated 5Ag in different sizes. Our measurements revealed a positive correlation between the contact area and the maximum adhesive force. However, we also observed an edge‐leaking behavior for relatively smaller sample. The theoretical adhesive force is estimated via the equation below^[^ ^24^ ^]^

(7) $$F=P_{d}\times A\times \;\left(1-P\right)$$

whereas the *F*, *P*_d_, *A*, and *P* represent the adhesive force, the pressure difference between the outside and inside of chitosan composite, the geometric surface area, and the porosity (≈84.3%). Accordingly, the theoretic adhesive force for a 5 × 3.5 × 0.2 cm^3^ chitosan composite is 21.9 N. The reasons that we only recorded 9.8 N (0.56 N cm^−2^) in adhesive force are; (1) we utilized a passive suction cup so continuous vacuum was not available to maintain the same magnitude of negative pressure^[^ ^25^ ^]^ and (2) the exact contacting area between the sample and the neighboring surface was also affected by humidity and local roughness.^[^ ^26^ ^]^

It is important to compare the adhesive force of our chitosan composite with what have been published in the literature. Table S2 (Supporting Information) lists the structures and their corresponding adhesive forces. For example, Geim et al. and Shan et al. prepared biomimetic gecko foot‐hair arrays and recorded an impressive adhesive force per unit area.^[^ ^27^ ^]^ Unfortunately, their fabrication steps were time‐consuming and complicated. Alternatively, Kessens et al. demonstrated an adhesive suction cup that maintained a decent adhesive force.^[^ ^28^ ^]^ However, a vacuum pump was necessary to keep the adhesive force intact for extended time. Additional suction cups were demonstrated and their adhesion forces were facilitated by externally imposed electric or magnetic field.^[^ ^26^, ^29^ ^]^ In contrast, our chitosan composite (5Ag) revealed an impressive adhesive force even with a partial physical contact. Figure 7c displays the photographs showing our sample with sufficient adhesive force toward attachments with different curvatures. This ability was attributed to the vertically‐aligned independent channels and structural flexibility that accommodated readily to surface with uneven morphologies. It is noted that we are the first group to introduce chitosan composites for adhesive applications such as object manipulation and negative pressure wound dressing.

## 3 Conclusion

A chitosan composite consisting of chitosan, PEG, and nanoparticles of Ag_2_O and Ag was successfully synthesized by an EPD approach. The chitosan composite demonstrated a honeycomb‐like microstructure in which independent pore channels of 488 µm in diameter and 2 mm in length were aligned vertically with an effective porosity of 84%. This unique structure was obtained by a delicate balance between the chemical constituents and EPD processing parameters. Comprehensive material characterization was carried out and detailed formation mechanism was discussed and videotaped. The chitosan composite revealed a water uptake of 2100% and a desirable two‐stage silver release profile. Due to its straight pore channels, the chitosan composite allowed a fast mass transport over that of commercially available gauze. In addition, the chitosan composite demonstrates an adhesive force of 9.8 N (0.56 N cm^−2^), and was validated to maintain necessary adhesive force toward different curved surface.

## 4 Experimental Section

### Preparation of EPD Solution

First, 1 g chitosan (degree of deacetylation: >90%; molecular weight: 100–130 kDa; Charming and Beauty Co., Ltd.) was dissolved in an aqueous solution containing 0.5 mL acetic acid (CH_3_COOH; ≧99.8%; Sigma Aldrich) and 49.5 mL deionized water. The mixture was stirred for 5 h at 70 °C to obtain a homogeneous solution. Afterward, the chitosan solution was cooled to 25 °C, followed by the addition of different amounts of 100 × 10^−3^ m silver nitrate (AgNO_3_; ≧99.9%; Alfa Aesar) aqueous solution. The resulting AgNO_3_ concentration in the mixture was 4.3, 12.9, and 21.5 × 10^−3^ m, respectively. After stirring for 10 min, 7 g polyethylene glycol (PEG; MW = 600 Da; SHOWA) was added and stirred for 24 h at 25 °C to form the electrophoresis solution. The color for the mixture was changed from light yellow to purple in 24 h, indicating the formation of Ag NPs from the reduction of Ag^+^ by PEG. Prior to the EPD, 1 mL of H_2_O_2_ was added into the electrophoresis solution to reduce the H_2_ bubble formation from subsequent EPD process.

### EPD Fabrication of Chitosan/PEG/Ag_2_O‐Ag NPs Composite

The EPD was carried out in a potentiostatic mode using a two‐electrode cell in which a Pt foil (5 × 3.5 cm^2^) and a stainless steel plate (5 × 3.5 cm^2^) were used as the counter and working electrode, respectively. The voltage was kept at −10 V and the distance between the electrodes was 3 cm, resulting in an effective electric field of −3.3 V cm^−1^. The EPD lasted for 10 min, and the sample was removed from the working electrode by peeling and was kept in a freezer at −20 °C for 48 h, followed by a freeze‐drying process at −80 °C for 72 h.

### Materials Characterization

A SEM (JEOL JSM‐6700F) was used to observe the chitosan composite for pore size distribution and surface morphology. X‐ray Diffraction (XRD; Bruker D2 Phaser) with a Cu K*α* radiation (*λ* = 1.54 Å) was used to determine the crystallinity of chitosan and Ag_2_O‐Ag NPs in the chitosan composite, and the diffraction angle (2*θ*) was set from 10° to 60°. To explore the thermal stability of chitosan composite, thermogravimetric analysis (TGA; TA Instruments TGA Q500) was conducted with a heating rate of 10 °C min^−1^ from 25 to 700 °C in air. XPS (Thermo Fisher Scientific ESCALAB Xi^+^, Kratos Axis Ultra DLD) was used to elucidate the chemical nature of constituents in chitosan composite with a probing depth below 5 nm and a monochromatic X‐ray source (Al anode).

### Water Uptake Experiment

For experiments on water uptake, samples of pristine chitosan and chitosan composites were cut to 1 × 1 cm^2^ and weighed in the dry state (*W*_d_). Afterward, they were soaked in deionized water for a predetermined time, and were retrieved with surface water removed to measure their net weight in a wet state (*W*_w_). The water uptake ratio was determined by (*W*_w_ − *W*_d_)/*W*_d_ at 100%. To obtain the Ag release profile, the chitosan composites were cut to 1 × 2 cm^2^ (9–11 mg) and soaked in 10 mL phosphate buffer saline (1X PBS) at 37 °C. At a predetermined interval, 2 mL aliquot was collected and refilled with fresh PBS by the same amount. The collected samples were analyzed by an inductively coupled plasma optical emission spectrometer (ICP‐OES; Perkin Elmer OPTIMA 2000DV) to determine the concentration of Ag^+^ in PBS.

### Mass Transport Experiment

In mass transport experiments, the chitosan composite (5Ag) and gauze were cut to 1 × 1 × 0.2 cm^3^, and immersed in deionized water for 10 min. Next, they were placed atop a quartz cuvette containing 4.3 mL deionized water. Subsequently, a droplet of 100 µL was placed onto the sample, and the deionized water in the cuvette was analyzed by an UV–vis spectrometer (Agilent Technologies Cary 60) for 60 min. The resulting UV–vis spectra were recorded from 600 to 200 nm with a scan rate of 300 nm min^−1^. The droplet contained either 8 mg mL^−1^ Disperse Red 13 (≈25%, Aldrich Chemical Company, Inc.) or 48 mg mL^−1^ ZnO powders (Fisher Scientific).

### Adhesive Force Analysis

The maximum adhesive force was determined using a similar method as described in Okuno et al.^[^ ^24^ ^]^ The 5Ag was fabricated directly on a stainless steel foil in a wet state (5 × 3.5 × 0.2 cm^3^), and the backside of the stainless steel was glued to a glass cylinder via a double‐sided tape. Next, the sample was vacuumed for two time at −80 kPa for 5 min, and returned to the ambient atmosphere for following suction tests. The adhesive force was recorded by loading different weights for 10 s, and the measurement was considered complete after 10 successful trials in a row. The maximum adhesive force was defined by the largest weight sustained by the sample.

### Statistical Analysis

1. Preprocessing of data: N/A.

2. Data presentation: mean and mean ± SD.

3. Sample size for each statistical analysis: All the statistic graphs presented with error bars contain 3 replicates at each point.

4. Software used for statistical analysis: Excel.

## Conflict of Interest

The authors declare no conflict of interest.

## Supporting information

Supporting Information

Supplemental Video 1

Supplemental Video 2

Supplemental Video 3

## Data Availability

The data that support the findings of this study are available from the corresponding author upon reasonable request.;
