# Supplementary material for: Bubble‐Channeling Electrophoresis of Honeycomb‐Like Chitosan Composites
Source: Adv Sci (Weinh). 2022 Sep 30;9(32):2203948. doi: 10.1002/advs.202203948 (PMC9661845; doi:10.1002/advs.202203948)
Supplement: Supplementary file 1 — Supporting Information [file ADVS-9-2203948-s002.pdf]

ADVANCED SCIENCE
Open Access

Supporting Information

for *Adv. Sci.*, DOI 10.1002/advs.202203948

Bubble-Channeling Electrophoresis of Honeycomb-Like Chitosan Composites

Bo-Han Huang, Li-Jie Chen, Yu-Jie Chiou, Grace Whang, Yunkai Luo, Yichen Yan, Kung-Hwa Wei, Ximin He, Bruce Dunn and Pu-Wei Wu**

Supporting Information

Bubble-channeling electrophoresis of honeycomb-like chitosan composites

Bo-Han Huang,[1] Li-Jie Chen,[1] Yu-Jie Chiou,[1] Grace Whang,[2] Yunkai Luo,[2] Yichen Yan,[2] Kung-Hwa Wei,[1] Ximin He,[2] Bruce Dunn,[2,] and Pu-Wei Wu[1,]**

[1]Department of Materials Science and Engineering
National Yang Ming Chiao Tung University, Hsinchu 300, Taiwan
E-mail: ppwu@nycu.edu.tw

[2]Department of Materials Science and Engineering
UCLA, Los Angeles, CA 90095, USA
Email: bdunn@ucla.edu

This PDF file includes:

Figure S1 to S10

Table S1 and S2

Other Supporting Materials:

Video S1 to S3

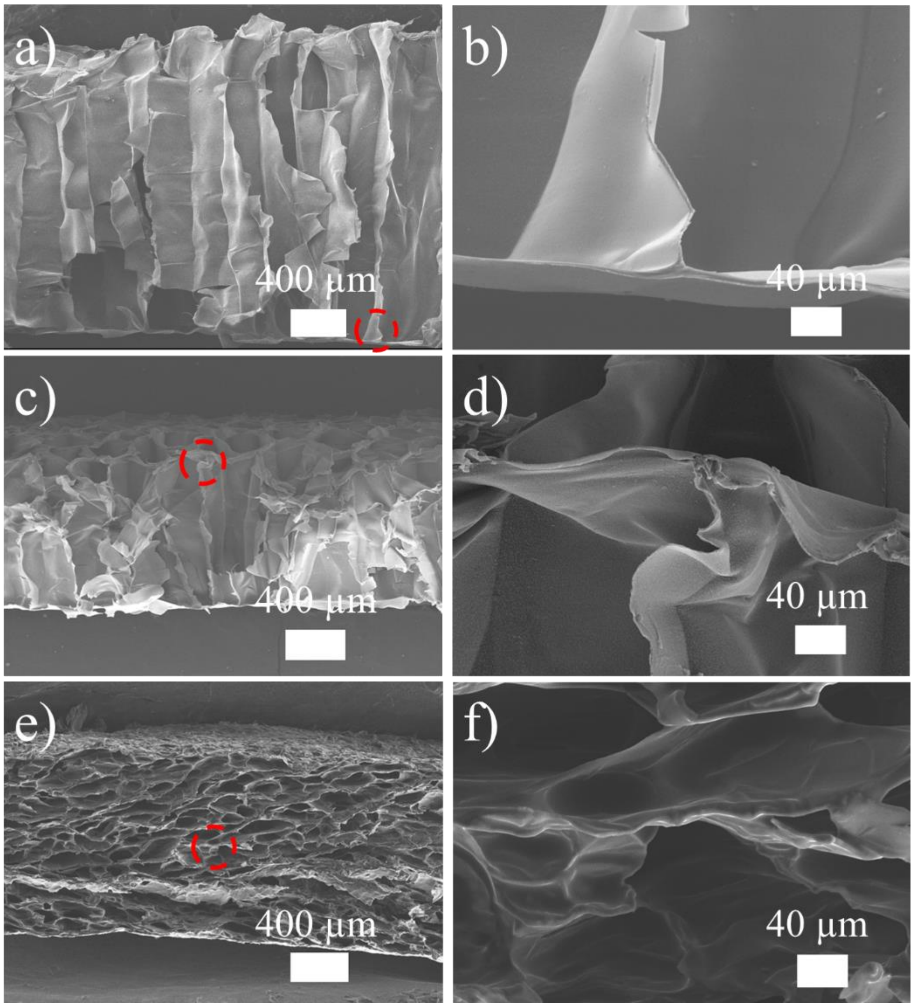

Figure S1. a) The cross-sectional SEM image of 5Ag (EPD). b) The magnified cross-sectional SEM image of 5Ag (EPD) from the red dash circle in a). c) The cross-sectional SEM image of 5Ag (oven-drying). d) The magnified cross-sectional SEM image of 5Ag (oven-drying) from the red dash circle in c). e) The cross-sectional SEM image of 5Ag (casting). f) The magnified cross-sectional SEM image of 5Ag (casting) from the red dash circle in e).

For EPD of 5Ag electrophoresis solution, the chitosan molecules were precipitated on the cathode along with the deposition of PEG and Ag/Ag_2O nanoparticles. The deposit was a dense film that was peeled off readily in a wet state. The dense film contained very few water so the formation of water crystals upon freeze-drying became rather negligible, as shown in Figure S1. Figure S1a displays a SEM cross-sectional view of 5Ag. A larger magnification view is displayed in Figure S1b. It is clear that the formation of ice crystal was not observed. To further validate there was negligible residual water in our case, we also prepared a sample that

underwent oven-drying instead of freeze-drying. The sample was prepared using the 5Ag electrophoresis solution undergoing the same EPD process. Subsequently, the sample was left in an oven at 50℃ for 72 h (the sample is denoted as the 5Ag (oven-drying)). To minimize any deformation during the oven-drying step, a glass bottle of 300 g was positioned atop the sample. The resulting cross-sectional images are displayed in Figure S1c,d. It is noted that the thickness for 5Ag (oven-drying) was reduced due to the weight of the glass bottle, but the honeycomb-like microstructure was still retained. Again, we did not observe any trace of ice crystals and its morphology appeared similar to that of 5Ag derived from EPD. Lastly, we also prepared a sample derived from a straightforward casting of 5Ag electrophoresis solution (the sample is denoted as the 5Ag (casting)). Apparently, the sample from the casting route, followed by the freeze-drying step, revealed a porous structure with a random pore size distribution, as displayed in Figure S1e,f. These random pores were likely caused by the formation of ice crystals. It is noted that the morphology from our 5Ag (casting) was similar to those reported in the literature from the casting route.[1]

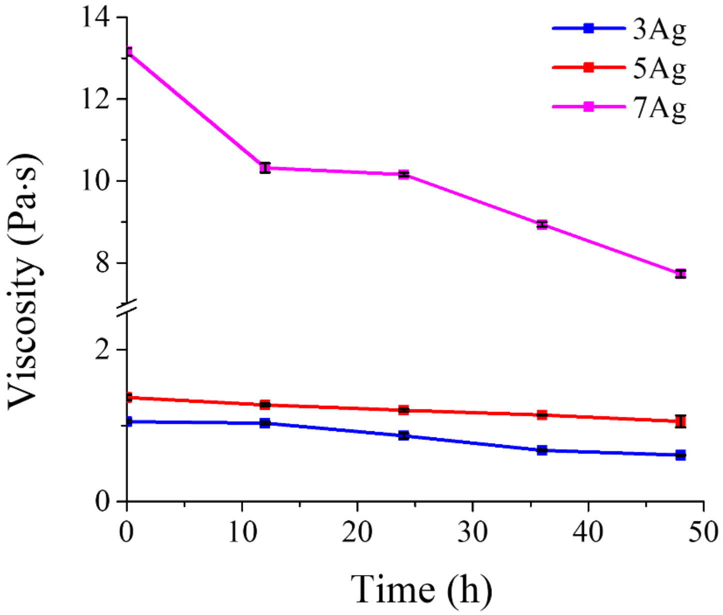

Figure S2. The viscosity of 3Ag, 5Ag, and 7Ag as a function of time. The error bar represents its statistical standard deviation. Three measurements are taken for each data point.

Additional rheology analysis was carried with samples of 3Ag, 5Ag, and 7Ag (the composition for 7Ag contained chitosan 1.43% (w v^{-1}), PEG 10% (w v^{-1}), $AgNO_3$ 30.1 mM, and H_2O_2 2.2% (w v^{-1})). These rheology experiments were conducted using a rheometer (TA Instrument, Discovery HR 10) to obtain the viscosity data at the 0th, 12th, 24th, 36th, and 48th h, respectively. Each measurement was repeated for three times to obtain the average and standard deviation. The resulting viscosity profiles are displayed in Figure S2. As shown, for 5Ag its viscosity at the 0th h reached the highest value of 1.37±0.03 Pa·s, which was attributed to the presence of Ag^+ interacting with chitosan molecules. As time progressed, the viscosity was reduced steadily at a constant rate of -0.0064 Pa·s h^{-1}. This phenomenon was believed to be caused by the formation of Ag nanoparticles that reduced the magnitude of interaction between the Ag^+ and chitosan molecules.[2] For 3Ag, the highest value of viscosity was 1.08 Pa·s, which was occurring at the 0th h. In addition, a similar pattern of decreasing viscosity with time was observed. Since its Ag^+ concentration was smaller than that of 5Ag, the viscosity for 3Ag was consistently lower than that of 5Ag (due to a relatively weaker interaction of Ag^+ and chitosan molecules). For 7Ag, the highest value of the viscosity was 13.24 Pa·s, which was occurring at the 0th h. Notably, this

value was 10 times greater than the viscosity of 5Ag at the 0th h. As expected, the 7Ag contained excess presence of Ag^+ that engendered a stronger interaction between Ag^+ and chitosan molecules. As a result, the solution became semi-gel-like quickly, leading to unstable results in following EPD process.

It is noted that in the 5Ag electrophoresis solution, the magnitude of viscosity change was merely 0.001 Pa·s during the duration of electrophoresis (10 min). Therefore, we realized that the EPD was essentially performed at a constant viscosity. This helped to explain the structural uniformity of chitosan composite in our study.

An additional point is that among these three samples, the viscosity was decreasing slowly as time progressed. It is because once the Ag^+ was reduced to Ag nanoparticles, the magnitude of interaction between the Ag^+ and chitosan molecules became weaker. As a result, the viscosity was reduced and the sample revealed a “self-collapsing” behavior. Similar phenomenon has been reported in the literature.[3]

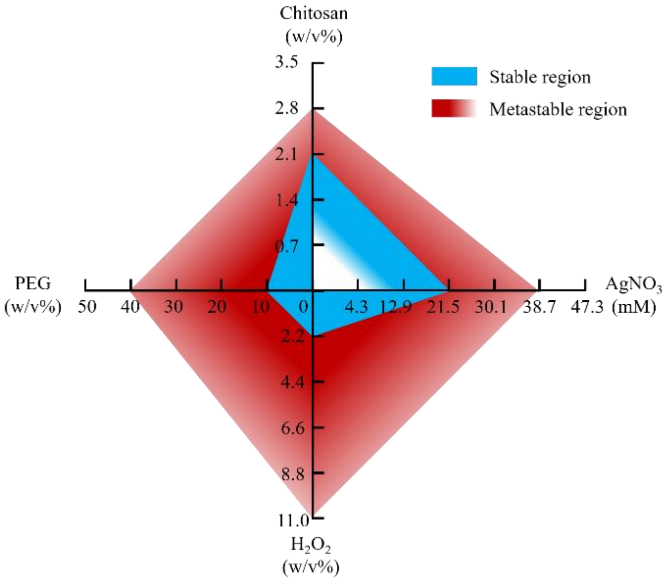

Figure S3. Possible chemical composition of electrophoresis solution for the fabrication of chitosan composites. The composition in the area highlighted in blue is able to form stable samples, while the composition in the area highlighted in red forms metastable samples. The composition in the area highlighted in white is not able to form desirable chitosan composite.

Table S1. Feasibility of honeycomb-like chitosan composites with different compositions.

ID	Chitosan % (w v^{-1})	PEG % (w v^{-1})	$AgNO_3$ (mM)	H_2O_2 % (w v^{-1})	Honeycomb-like Composites?
**5Ag (0.5CHI)**	0.7	10	21.5	2.2	Yes
**5Ag**	1.43	10	21.5	2.2	Yes
**7Ag**	1.43	10	30.1	2.2	Metastable
**9Ag**	1.43	10	38.7	2.2	Metastable
**11Ag**	1.43	10	47.3	2.2	No (Gelling)
**5Ag (1.5CHI)**	2.1	10	21.5	2.2	Yes
**5Ag (2CHI)**	2.8	10	21.5	2.2	Metastable
**5Ag (2.5CHI)**	3.5	10	21.5	2.2	No (Gelling)
**5Ag (0PEG)**	1.43	0	21.5	2.2	Yes
**5Ag (2PEG)**	1.43	20	21.5	2.2	Metastable
**5Ag (3PEG)**	1.43	30	21.5	2.2	Metastable
**5Ag (4PEG)**	1.43	40	21.5	2.2	Metastable
**5Ag (5PEG)**	1.43	50	21.5	2.2	No (Gelling)
**5Ag ($0H_2O_2$)**	1.43	10	21.5	0	Yes
**5Ag ($2H_2O_2$)**	1.43	10	21.5	4.4	Metastable
**5Ag ($3H_2O_2$)**	1.43	10	21.5	6.6	Metastable
**5Ag ($4H_2O_2$)**	1.43	10	21.5	8.8	Metastable
**5Ag ($5H_2O_2$)**	1.43	10	21.5	11	Metastable
**5Ag ($6H_2O_2$)**	1.43	10	21.5	13.2	No (Too Dilute)

The electrophoresis solution with different compositions were carefully evaluated for their possibilities in fabricating chitosan composites with desirable vertically aligned pore channels. The results are depicted in Figure S3 and Table S1 for better understanding. In Figure S3, the area highlighted in blue includes the compositions that were able to form desirable chitosan composites. The area highlighted in red includes the compositions that formed metastable chitosan composites. In the metastable state, the electrophoresis solution became viscous or gel-like, and thus vertically aligned independent pore channels were unable to form uniformly, i.e., the yield was poor. For the area highlighted in white, the concentration of Ag^+ or chitosan was too low for a sufficient deposition rate so the formation of chitosan composites with vertically aligned pore channels was not possible.

In our electrophoresis solution, the protonated chitosan, Ag^+ ions and Ag nanoparticles were the primary constituents. According to our results, it is necessary for the Ag^+ concentration to reach 12.9 mM (3Ag) in order to produce a honeycomb-like structure after EPD process. However, once the Ag^+ concentration reached above 21.5 mM (5Ag), the electrophoresis solution became a semi-gel-like state that the EPD was not able to proceed.

The concentration of chitosan would also affect the EPD rate. In our observation, the lowest concentration of chitosan necessary for the formation of honeycomb-like structure was 0.7% (w v^{-1}), as shown in Figure S3. Any electrophoresis solution with chitosan concentration less than 0.7% (w v^{-1}) was unable to produce a honeycomb-like structure due to its subdued amount in the electrophoresis solution. In addition, we also observed that the deposition rate was decreasing with increasing chitosan concentration. However, the electrophoresis solution turned to gel-like, and thus we were unable to conduct the EPD process once the chitosan concentration reached above 2.8% (w v^{-1}). It is because the interaction between Ag^+ and chitosan became excessive.

The function of H_2O_2 in the electrophoresis solution is to remove excess H_2 bubbles that were formed during EPD. Thus, a honeycomb-like structure was obtained readily with the addition of H_2O_2. However, we observed that even without the addition of H_2O_2, samples with honeycomb-

like structure were still be formed, albeit in a narrower composition range. On the other hand, the honeycomb-like structure was not observed once the H_2O_2 concentration reached over 11% (w v^{-1}). It is because that the over-dilution of the electrophoresis solution (the H_2O_2 was 30 wt% so excess H_2O_2 indicated that additional water was added, which effectively diluted the overall electrophoresis solution). The overall composition range for honeycomb-like structure is displayed in Figure S3.

In our electrophoresis solution, the PEG was served as a stabilizer because the PEG was not participating in any chemical reaction with other constituents in the electrophoresis solution during EPD. Instead, the PEG was present to reduce the water electrolysis rate during the EPD so the honeycomb-like structure was able to be formed more easily. It is noted that from our experiences, the honeycomb-like structure was able to be formed with electrophoresis solution without the addition of PEG. However, the resulting structural integrity appeared notably poor. It is noted that from our experiments, the maximum PEG concentration for desirable honeycomb-like structure was 40% (w v^{-1}), and with a larger PEG concentration, the electrophoresis solution became viscous, and was thus unable to form the honeycomb-like structure, as displayed in Figure S3.

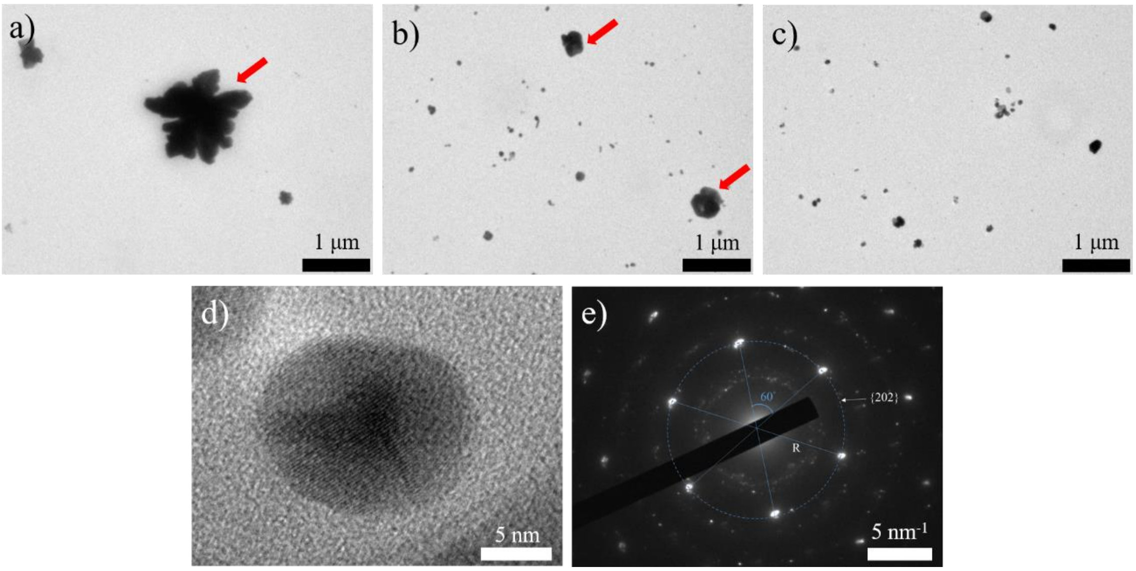

Figure S4. TEM observation of Ag NPs in electrophoresis solution.

Ag NPs in electrophoresis solution were observed by transmission electron microscopes (TEM; JEOL JEM2000FXⅡ, JEOL JEM2100) to determine their average size and standard deviation. In our electrophoresis solution, the hydroxyl groups in PEG were able to reduce the Ag^+,[4] and as time progressed, the solution became darker with the formation of Ag NPs. Figure S4a-c display low magnification TEM images for 1Ag, 3Ag, and 5Ag. Apparently, the size of Ag NPs was decreased with increasing $AgNO_3$ concentration; for 1Ag, 3Ag, and 5Ag, their resulting Ag NP size was 105±71, 95±36, and 67±24 nm, respectively. In addition, the Ag NPs in 1Ag and 3Ag revealed a notable aggregation. We surmised that at a relatively low Ag^+ concentration, a smaller number of Ag nuclei was formed so the competition for growth was less intense. In contrast, with a relatively high Ag^+ concentration, both nucleation and growth were occurring simultaneously so the average Ag NP size became smaller. Figure S4d displays the high magnification TEM image of 5Ag. Apparently, there appeared clear fringes which were often observed for metallic NPs. Figure S4e displays the corresponding TEM diffraction pattern of 5Ag. As shown, those primary diffraction spots were attributed to the {202} of fcc lattice (B=[111]), as evidenced by a perfect intersection angle of 60°. Since the radius for the reciprocal circle (R) was 6.93 nm^{-1}, the resulting d spacing ($R=d^{-1}$) was 1.44 Å, a value that agreed well with the theoretical spacing of {202} in Ag.

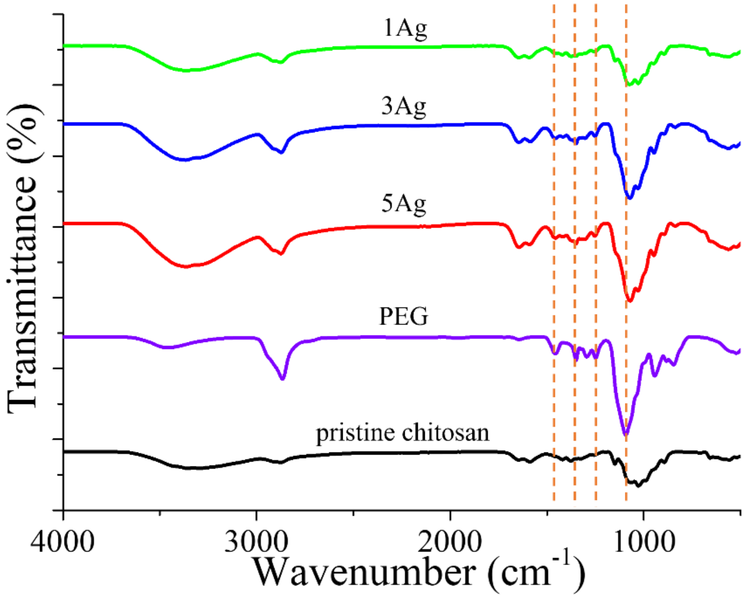

Figure S5. FT-IR analysis of 1Ag, 3Ag, 5Ag, PEG, and pristine chitosan.

An attenuated total reflection Fourier-transformed infrared spectrometer (FT-IR; PerkinElmer Spectrum 100) was employed to identify the nature of chemical bonding in chitosan and PEG, as well as chitosan composites of 1Ag, 3Ag, and 5Ag. The samples were subjected to FT-IR scans 32 times in wavenumbers between 4,000 and 400 cm^{-1} with a resolution of 4 cm^{-1}, and the resulting profiles are displayed in Figure S5. For pristine chitosan, the FT-IR spectrum is consistent with what was reported in earlier literature in which the signal at 1,150 cm^{-1} is assigned to the vibration mode of glycosidic bonding (-C-O-C-), and the signal at 1,024 cm^{-1} is caused by the C-O stretching.[5] In addition, the signal at 1,590 cm^{-1} is associated with the amide II band of NH-bending and the signal around 1,322 cm^{-1} is due to the amide III band.[5,6] For PEG, the signals around 1,094 cm^{-1} and 1,279 cm^{-1} are caused by the C-O-H stretching and the signals around 1,343 cm^{-1} and 1,464 cm^{-1} are attributed to the C-H bending.[7] For 1Ag, 3Ag, and 5Ag, they revealed signals from those of chitosan and PEG validating our chitosan composites are indeed consisted of chitosan and PEG.

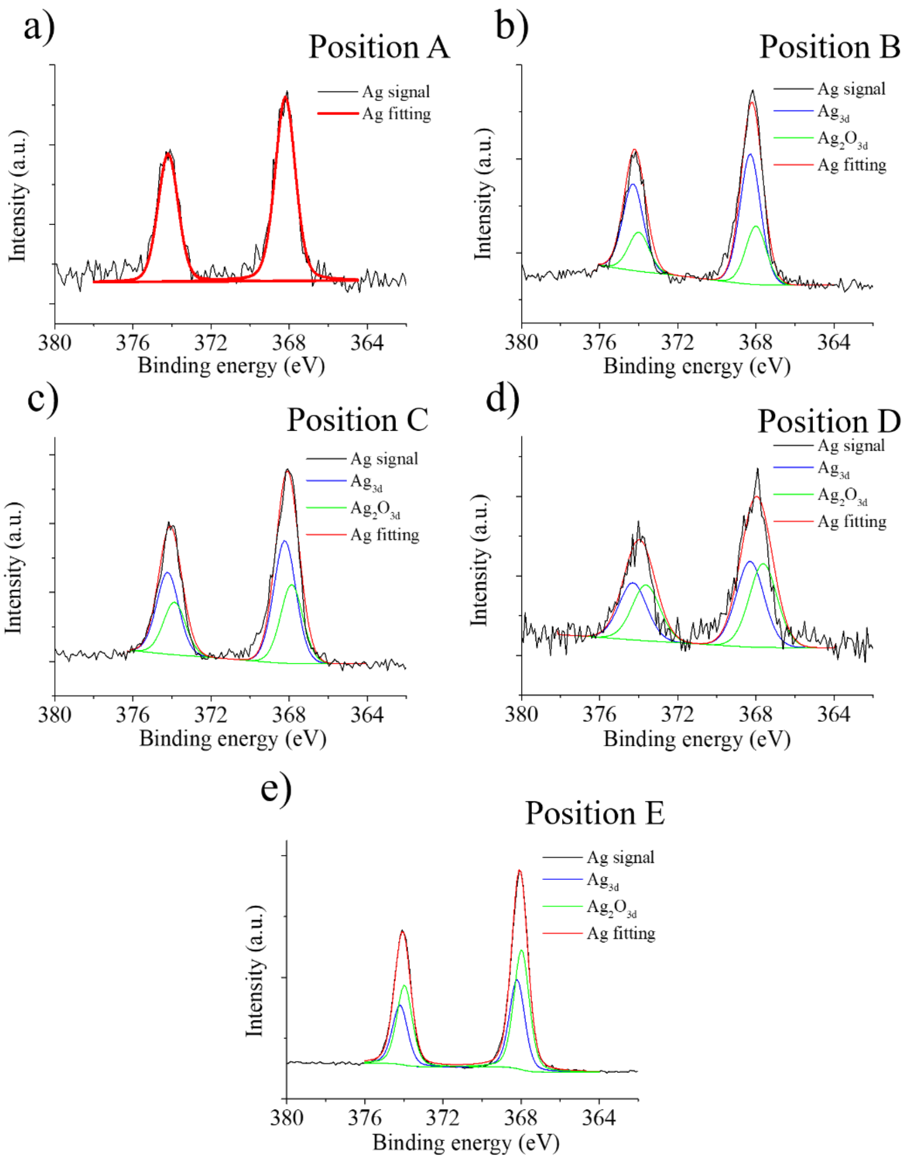

Figure S6. Cross-sectional XPS Ag(3d) analysis of 5Ag and their curve fitting results.

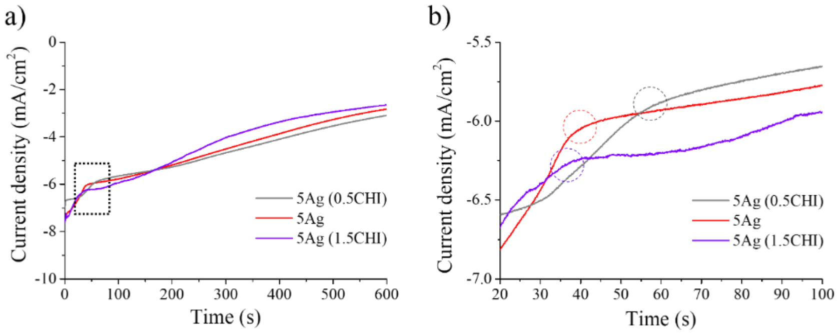

Figure S7. The current profiles of 5Ag, 5Ag (0.5CHI), and 5Ag (1.5CHI) during EPD; a) for the entire 600 s and b) for the first 100 s.

0.5, 1, and 1.5 g chitosan (degree of deacetylation: >90%; molecular weight: 100~130 kDa; Charming and Beauty Co., Ltd.) were dissolved in an aqueous solution containing 0.5 mL acetic acid (≧99.8%, Sigma Aldrich) and 49.5 mL deionized water. The mixtures were stirred for 5 h at 70℃ to obtain homogeneous solutions. Afterward, the solutions were cooled to 25℃, followed by the addition of 100 mM $AgNO_3$ aqueous solution. The resulting $AgNO_3$ concentration in the mixture was 21.5 mM. After stirring for 10 min, 7 g polyethylene glycol (PEG; MW=600 Da; SHOWA) was added and stirred for 24 h at 25℃ to form the electrophoresis solution. The solutions added with 0.5, 1, and 1.5 g chitosan were named 5Ag (0.5CHI), 5Ag, and 5Ag (1.5CHI), respectively.

The chitosan composites were fabricated via EPD in a potentiostatic mode. The EPD was carried out in a two-electrode cell in which a Pt foil (3.5×5 cm^2) and stainless steel (3.5×5 cm^2) were used as the counter and working electrode, respectively. 1 ml of H_2O_2 was added to the electrophoresis to remove the hydrogen bubbles from the water electrolysis reaction. The EPD voltage was kept at -10 V and the distance between the electrodes was 3 cm, resulting in an effective electric field of -3.3 V cm^{-1}. The EPD lasted for 10 min, and the chitosan composites (named 5Ag (0.5CHI), 5Ag, and 5Ag (1.5CHI)) in a wet state were detached from the working electrode by peeling and kept in a freezer at -20℃ for 48 h, followed by a freeze-drying process at -80℃ for 72 h.

Figure S7a displays the current profiles for 600 s of EPD for 5Ag (0.5CHI), 5Ag, and 5Ag (1.5CHI). Apparently, their current profiles were reasonably stable and the coulomb charge (the

area underneath the current profile) were similar. The coulomb charge of 5Ag (0.5CHI), 5Ag, and 5Ag (1.5CHI) was 48.61±1.01 C, 47.36±1.61 C, and 46.48±2.39 C, respectively. Figure S7b displays the magnified view of current profiles for the period between 20 and 100 s. It is noted that a larger chitosan concentration rendered an earlier transition stage (dashed circles), at which the deposition was dominated by hydrogen bubbles expansion (three-dimensional growth) to hydrogen bubbles elongation (one-dimensional growth). As a result, the Ag (1.5CHI) exhibited the smallest pore channel diameter, as evidenced in Figure S9.

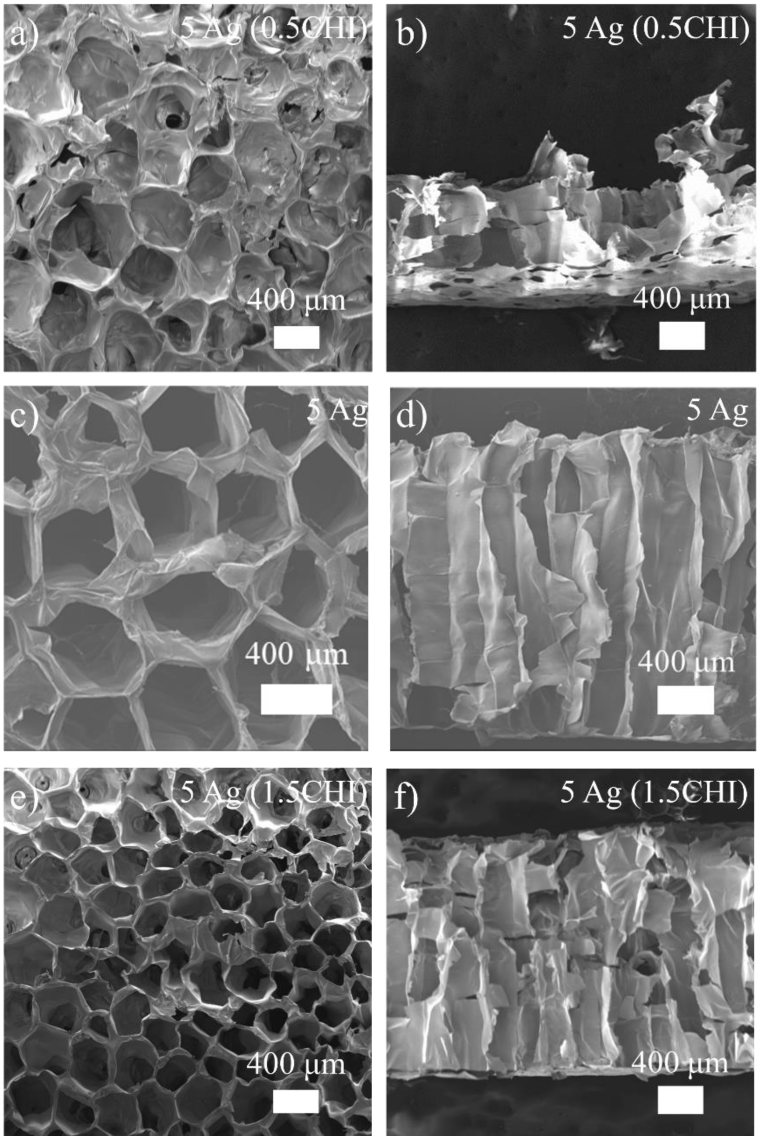

Figure S8. SEM images of 5Ag (0.5 CHI) in a) top view and b) cross-sectional view, 5Ag in c) top view and d) cross-sectional view, and 5Ag (1.5 CHI) in e) top view and f) cross-sectional view.

Figure S8 displays the SEM images of 5Ag (0.5CHI), 5Ag, and 5Ag (1.5CHI). From the top view images, shown in Figure S8a,c,e, the pore channel diameter became smaller with increasing chitosan concentration. From cross-sectional view in Figure S8b,d,f, it was observed that the thickness of 5Ag (0.5CHI) was the smallest because of its relatively low chitosan concentration. On the other hand, the thickness of 5Ag and 5Ag (1.5CHI) were rather similar because there was

a thickness limitation (~2 mm) in our EPD process. According to Figure 5c, the chitosan deposition is affected by the water transportation path as a thicker chitosan composite renders a longer water transportation route and thus, the water electrolysis is compromised. With a dwindling water electrolysis reaction, the chitosan deposition is inhibited. Thus, the thickness limitation for our chitosan composite is around 2 mm. This behavior was also supported by the slowly reduced current during EPD, as shown in Figure S7a.

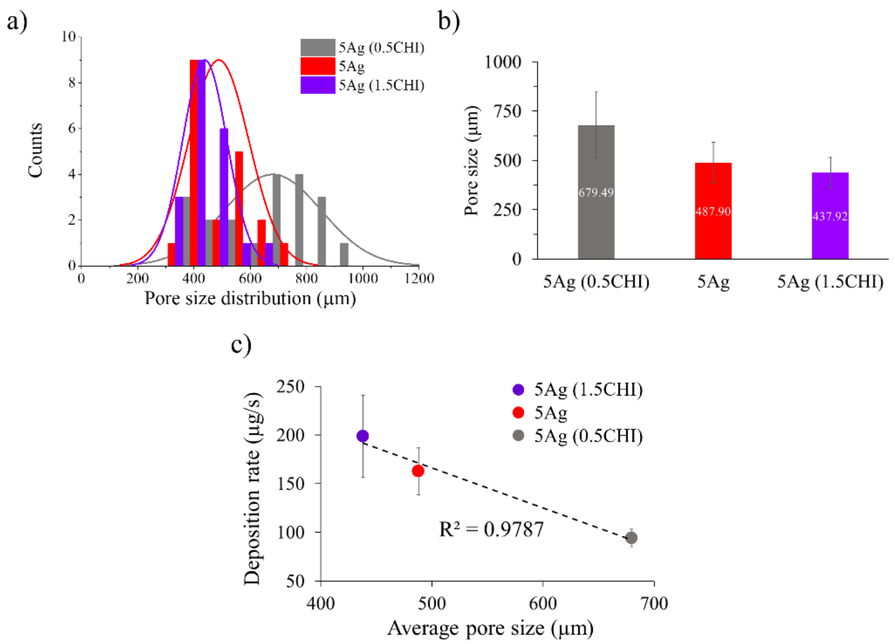

Figure S9. Analysis of 5Ag, 5Ag (0.5CHI), and 5Ag (1.5CHI); a) channel diameter distribution, b) average channel diameter, and c) comparison between deposition rate and average channel diameter.

The distribution in channel diameter for different chitosan composites is displayed in Figure S9a. Apparently, the 5Ag (1.5CHI) exhibited the narrowest distribution whereas the 5Ag (0.5CHI) exhibited the broadest. Figure S9b displays the average channel diameter for those samples. As expected, the channel diameter was inversely proportional to the concentration of chitosan. In Figure S9c, the deposition rate was found to be highly linear to the average channel diameter (R^2 = 0.9787). We rationalize that a faster deposition rate could immobilize the growing hydrogen bubbles and render the transition from bubble expansion to bubble elongation earlier.

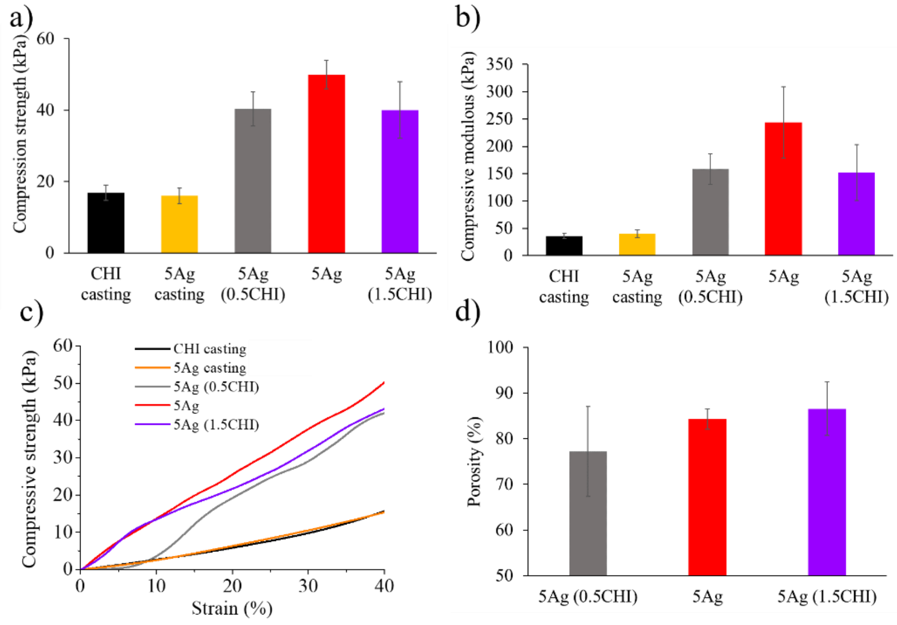

Figure S10. Analysis of mechanical properties of 5Ag, 5Ag (0.5CHI), and 5Ag (1.5CHI); a) compression strength, b) compressive modulus, c) strength-strain curve, and d) comparison of porosity.

The casting samples, denoted as the "CHI casting" and "5Ag casting", were fabricated by pouring 3 mL pristine chitosan and 5Ag electrophoresis solution into Petri dishes (6 cm in diameter) and keeping them in a freezer at -20℃ for 48 h, followed by a freeze-drying process at -80℃ for 72 h. The mechanical tests were measured by a Dynamic Mechanical Analyzer (DMA; TA Instrument DMA850) in a compression mode. The strain ramping rate was 20 % min^{-1}, and each sample was measured five times to obtain the average compression strength (defined at 40% strain) and the compressive modulus.[8] The compressive modulus is defined by the maximum slope of the stress-strain curve in the strain range between 0% and 20%.[9] The porosity was determined by a liquid displacement method.[1a] First, the samples were cut into 5 pieces of 1×1 cm^2 and the dry weight (W_d) and outer surface volume (V_d) were measured. Second, the samples were immersed in ethanol (ρ of 0.7893 g cm^{-3}) for 24 h and the wet weight (W_e) was recorded. Next, the porosity could be obtained by the following equation.

$$P\ (\%) = \frac{W_e - W_d}{\rho V_d}$$

Figure S10a displays the compression strength among these samples. Apparently, the chitosan composites demonstrated better strength over that of casting samples. It is realized that the presence of orderly vertical pore channels in a honeycomb-like microstructure engenders a stronger structure than a disorderly microporous one.[10] The compressive modulus, shown in Figure S10b, revealed a similar trend. As expected, the casting samples exhibited a smaller modulus than those of chitosan composites due to the lack of structural support. The resulting strength-strain curves are displayed in Figure S10c. Among them, the 5Ag revealed a larger slope as compared to that of 5Ag (0.5CHI) because the latter had a wide range of channel diameter distribution. Interestingly, the 5Ag (1.5CHI) behaved differently from 5Ag despite both samples had similar channel diameter distribution. Thus, we carried out further measurements in the porosity, as shown in Figure S10d. As expected, the 5Ag (1.5CHI) was found to possess the highest porosity which inevitably led to the deterioration in its mechanical property.

Table S2. Comparison of different adhesive forces and mechanisms reported in the literature.

	Adhesion structures	Maximum adhesive force (N)	Adhesive force (N cm^{-2})	Partial adhesion	Vacuum support	Electric or magnetic field
Geim *et al.*[11]	biomimetic gecko foot-hair array	3.00	3.00	Yes	No	No
Shan *et al.*[12]	biomimetic gecko foot-hair array	2.01	2.06	Yes	No	No
This work	honeycomb structure	9.80	0.56	Yes	No	No
Guo *et al.*[13]	soft pneumatic grippers with electroadhesion	10.00	~0.28	Yes	Yes	Yes
Kuwajima *et al.*[14]	suction cup actuated by electrohydrodynamics	0.028	0.064	No	Yes	Yes
Follador *et al.*[15]	octopus inspired suction cup	~2.40	0.60	No	Yes	Yes
Hu et al.[16]	suction cup with magnetic adhesion	0.49	0.045	No	No	Yes
Okuno *et al.*[17]	suction cup with electroadhesion	12.25	2.71	No	Yes	Yes
Kessens *et al.*[18]	suction cup array	12.50	8.12	No	Yes	No
Tsukagoshi *et al.*[19]	hybrid suction cup	80.00	~3.37	No	Yes	No

References

[1] a) Z. G. Wu, W. Zhou, W. J. Deng, C. L. Xu, Y. Cai, X. Y. Wang, *ACS Appl. Mater. Interfaces* **2020**, 12 (18), 20307; b) S. K. Samal, M. Dash, F. Chiellini, X. Wang, E. Chiellini, H. A. Declercq, D. L. Kaplan, *RSC Adv.* **2014**, 4 (96), 53547.

[2] a) M. Kozicki, M. Kolodziejczyk, M. Szynkowska, A. Pawlaczyk, E. Lesniewska, A. Matusiak, A. Adamus, A. Karolczak, *Carbohydr. Polym.* **2016**, 140, 74; b) H. Huang, Q. Yuan, X. Yang, *Colloids Surf. B* **2004**, 39 (1-2), 31.

[3] a) B. Mutharani, P. Ranganathan, S. M. Chen, *Int. J. Biol. Macromol.* **2019**, 124, 759; b) R. P. Ramasamy, S. M. Maliyekkal, *New J. Chem.* **2014**, 38, 63.

[4] C. Luo, Y. Zhang, X. Zeng, Y. Zeng, Y. Wang, *J. Colloid Interface Sci.* **2005**, 288, 444.

[5] a) Z. Zhang, X. Cheng, Y. Yao, J. Luo, Q. Tang, H. Wu, S. Lin, C. Han, Q. Wei, L. Chen, *J. Mater. Chem. B* **2016**, 4, 7584; b) F. Gebhardt, S. Seuss, M. C. Turhan, H. Hornberger, S. Virtanen, A. R. Boccaccini, *Mater. Lett.* **2012**, 66, 302; c) J. Kumirska, M. Czerwicka, Z. Kaczynski, A. Bychowska, K. Brzozowski, J. Thoming, P. Stepnowski, *Mar. Drugs* **2010**, 8, 1567.

[6] T. Jiang, Z. Zhang, Y. Zhou, Y. Liu, Z. Wang, H. Tong, X. Shen, Y. Wang, *Biomacromolecules* **2010**, 11, 1254.

[7] a) B. Chieng, N. Ibrahim, W. Yunus, M. Hussein, *Polymers* **2013**, 6, 93; b) W. S. Iqbal, K. L. Chan, *J. Pharm. Sci.* **2015**, 104, 280.

[8] S. Jana, S. J. Florczyk, M. Leung, M. Q. Zhang, *J. Mater. Chem.* **2012**, 22, 6291.

[9] R. J. Kane, H. E. Weiss-Bilka, M. J. Meagher, Y. Liu, J. A. Gargac, G. L. Niebur, D. R. Wagner, R. K. Roeder, *Acta Biomater.* **2015**, 17, 16.

[10] Q. C. Zhang, X. H. Yang, P. Li, G. Y. Huang, S. S. Feng, C. Shen, B. Han, X. H. Zhang, F. Jin, F. Xu, T. J. Lu, *Prog. Mater. Sci.* **2015**, 74, 332.

[11] A. K. Geim, S. V. Dubonos, I. V. Grigorieva, K. S. Novoselov, A. A. Zhukov, S. Y. Shapoval, *Nat. Mater.* **2003**, 2, 461.

[12] J. Shan, T. Mei, L. Ni, S. Chen, J. Chu, presented at 2006 1st IEEE International Conference on Nano/Micro Engineered and Molecular Systems, 18-21 Jan. **2006**.

[13] J. Guo, K. Elgeneidy, C. Xiang, N. Lohse, L. Justham, J. Rossiter, *Smart Mater. Struct.* **2018**, 27, 055006.

[14] Y. Kuwajima, H. Shigemune, V. Cacucciolo, M. Cianchetti, C. Laschi, S. Maeda, presented at *2017 IEEE/RSJ International Conference on Intelligent Robots and Systems (IROS)*, 24-28 Sept. **2017**.

[15] M. Follador, F. Tramacere, B. Mazzolai, *Bioinspir. Biomim.* **2014**, 9, 046002.

[16] X. C. Hu, Y. M. Fu, Y. D. Liu, B. H. Liu, S. X. Qu, *Adv. Mater. Technol.* **2021**, 6, 2100004.

[17] Y. Okuno, H. Shigemune, Y. Kuwajima, S. Maeda, *Adv. Mater. Technol.* **2019**, 4, 1800304.

[18] C. C. Kessens, J. P. Desai, *J. Mech. Robot* **2011**, 3, 045001.

[19] H. Tsukagoshi, Y. Osada, *Actuators* **2021**, 10, 50.
